# The Importance and Challenges of Implementing and Maintaining Biorepositories for High-Consequence Veterinary and One Health Pathogens in South-East Asia

**DOI:** 10.1089/apb.2023.0006

**Published:** 2024-02-28

**Authors:** Malinee Oyuchua, Jarunee Siengsanan-Lamont, Kim Khanh Le, Somjai Kamolsiripichaiporn, Jeeranan Areerob, Pacharee Thongkamkoon, Watthana Theppangna, Phouvong Phommachanh, Sothyra Tum, Barbara Johnson, Stuart D. Blacksell

**Affiliations:** ^1^ Mahidol-Oxford Tropical Research Medicine Unit, Faculty of Tropical Medicine, Mahidol University, Bangkok, Thailand.; ^2^ National Institute of Animal Health, Department of Livestock Development, Bangkok, Thailand.; ^3^ National Animal Health Laboratory, Department of Livestock and Fisheries, Ministry of Agriculture and Forestry, Vientiane, Lao PDR.; ^4^ National Animal Health and Production Research Institute, General Directorate for Animal, Health and Production, Phnom Penh, Cambodia.; ^5^ Biosafety and Biosecurity International, Merritt Island, Florida, USA.; ^6^ Centre for Tropical Medicine and Global Health, Nuffield Department of Medicine, Nuffield Department of Medicine Research Building, University of Oxford, Oxford, United Kingdom.

**Keywords:** biosecurity, biorepository management, pathogen inventory control, Southeast Asia, veterinary laboratories

## Abstract

**Introduction:**

Emerging infectious diseases pose a threat to public health and the economy, especially in developing countries. Southeast Asian veterinary laboratories handle numerous high-risk pathogens, making pathogen accountability crucial for safe handling and storage.

**Methods:**

Thirteen veterinary laboratories in Cambodia (*n* = 1), Lao People's Democratic Republic (*n* = 1), and Thailand (*n* = 11) participated in a study conducted between 2019 and 2020. Data were collected using a questionnaire, group discussions, and interviews.

**Conclusion:**

Significant gaps in biosecurity and biorepository management were recognized and discussed in the context of regional biosafety and biosecurity. Laboratories could use the findings and recommendations of the study to develop or improve their pathogen inventory and biosecurity systems. Governments play a significant role in setting standards and regulations and providing necessary support for laboratories to maintain inventory controls sustainably and have a very important role to play in ensuring biosafety and biosecurity compliance.

## Introduction

Concerns about emerging and re-emerging infectious diseases and their potential impacts on public health and the economy have increased over recent decades. In the past 25 years, outbreaks of zoonotic diseases were reported in Southeast Asia (SEA), including avian influenza,^1^ Nipah,^2^ anthrax,^3^ brucellosis,^4,5^ and the recent emergence of severe acute respiratory syndrome coronavirus 2 (SARS-CoV-2),^6,7^ the cause of the COVID-19 global pandemic.^8–10^

In addition, high-consequence animal diseases have been introduced to SEA, including African swine fever (ASF),^11^ African horse sickness (AHS),^12^ and lumpy skin disease,^13^ adding to the burden of foot and mouth disease, which remains an endemic in the region.^14,15^ These diseases threaten the public and economic health of the region^9,15–19^ and have forced veterinary laboratories to reorient their missions with increased activities in disease diagnosis, vaccine development, and pathogen research. There has been a significant increase in the volume and variety of biological materials, including infectious agents, being handled, stored, and archived in veterinary laboratories as a result.

Microorganisms are classified into risk groups (RG) based on their pathogenic characteristics. Microorganisms categorized as RG1 are the least harmful, with those in RG4 posing the highest risk.^20^ Generally, as the RG increases, there is a commensurate increase in the requirement for laboratory biosafety and biosecurity and inventory controls^20,21^; however, this also depends on the risks associated with the activities performed.

Many organisms handled by SEA veterinary laboratories are RG2 (e.g., leptospira, *Escherichia coli*) or RG3 (e.g., ASF virus, AHS virus). Ensuring sample and inventory security should be a common goal of all laboratories to minimize the risk of material loss, theft, intentional release or misuse by unauthorized personnel intending to cause harm. To achieve this, veterinary laboratories should implement systems for material accountability for high-risk samples while being manipulated, transferred, or stored at the facility.

Important components of a laboratory biosecurity system include personnel reliability, transport security, material accountability, emergency response, management, physical security, information security, and biosecurity awareness.^20,22,23^ This article focuses on two major components of laboratory biorisk management: inventory management and security.

Proper inventory control with accountability ensures safe and secure storage and handling of dangerous pathogens. The system should track information about biological materials, such as material type, collection date and time, container type, storage location, quality, and the owner or responsible person. It should be able to record various events related to the materials, such as freeze-thaw cycles, accessioning, removal from storage, inter-entity and intra-entity transfer, and disposal.

Currently, inventory systems may be paper-based systems, such as logbooks, or electronic-based systems, such as spreadsheets, databases, or specifically designed sample inventory management software. The choice of inventory system depends on many factors, including stock volume, laboratory facilities, and funding available within countries or organizations.^24,25^ Commercial inventory management software is available and implemented in many laboratories worldwide^10^ and often designed to manage biobanks that support a large and diverse biospecimen stock and associated data with essential security and audit trail features.^26^

Although guidelines and publications have been produced for establishing biospecimen and biobanking databases in many countries,^27–29^ only the Republic of Uganda^30^ had published their achievement of establishing a national database of pathogens during the period of our study. Their report also presented an intention of handling information collected from laboratories working with dangerous pathogens in a centralized, secure database, and this information could contribute to identifying and developing biosecurity measures in a country.^30^

It is acknowledged that a centralized database could pose a security risk since it would hold sensitive information about countries, including the locations and inventories of biological agents.^20^ To properly address this issue, it is necessary to integrate biorisk management practices in laboratories with cybersecurity controls. This approach identifies cyber risks and implements mitigation measures to reduce the likelihood of cyber-attacks, compromising the pathogen inventory database's confidentiality, integrity, or availability.^31^

To date, there are no international standard guidelines on biological inventory requirements. In SEA, several countries have implemented biosafety and biosecurity laws. Singapore and Thailand have established national regulations for handling, importing, exporting, and possession of biological agents and toxins. However, Lao People's Democratic Republic (Lao PDR), Cambodia, and Myanmar lack national legislation and guidelines for biosafety, biosecurity, and inventory control of dangerous pathogens and toxins.^32^

Our study investigates inventory systems for managing and storing pathogen stocks and infectious materials in government-owned veterinary laboratories staffed by government employees in Thailand, Lao PDR, and Cambodia. The collaborative projects aimed at enhancing biosafety and biosecurity capabilities in veterinary laboratories, identifying gaps and providing recommendations for future improvement and sustainment.

## Materials and Methods

Data were collected between 2019 and 2020 using a questionnaire, group discussion, and interview from the laboratory centers of Thailand (*n* = 11), whereas for Lao PDR (*n* = 1) and Cambodia (*n* = 1), data were only gathered from the national laboratories. Face-to-face group discussions were conducted at five Thai laboratory centers in October 2020, whereas the rest of the group discussions and interviews were completed online.

Each laboratory center had separate areas designated for specific disciplines such as Bacteriology, Virology, Parasitology, Microbiology, and Immunology. The veterinary laboratories' primary responsibilities were to provide animal disease diagnosis, quality control of animal products, and animal health research, except for one center where the primary responsibility was to test animal vaccines.

### Questionnaire

A biosecurity questionnaire was created in both Thai and English languages, containing eight main questions. Some questions were adapted from a previously published checklist.^33^ The questionnaire survey was emailed to Thai laboratory directors and completed by senior scientists or veterinarians. A follow-up group discussion with laboratory personnel from each center who worked with biological materials and pathogenic microorganisms was conducted on-site or online using Microsoft^®^ Teams.^34^

Thai staff were asked to rate their satisfaction with the inventory system, and identify issues and support required for improvement. In Laos and Cambodia, the English version of the survey was completed through online interviews and follow-up emails with laboratory directors and staff.

### Data and Statistical Analyses

The questionnaire survey and group discussion outcomes were summarized. Descriptive analyses and visualization of the data were performed using Microsoft Excel. All identified gaps were assessed using the survey answers, group discussion and interviews against the critical components of an inventory control recommended by WHO^20,35^ and Salerno and Gaudioso,^22^ and best practices for repositories described by the International Society for Biological and Environmental Repositories^36^ and other organizations.^37,38^

Recommendations against each identified gap were given based on the literature review and staff comments. The literature reviews of scientific journal publications^33,39^ and international guidelines^40^ on biosecurity about laboratory data and specimen inventory systems were conducted to select expert recommendations suitable for each gap. Laboratory staff comments were combined to create practical solutions specific to the local context.

## Results

### General Findings

**Figure 1.**
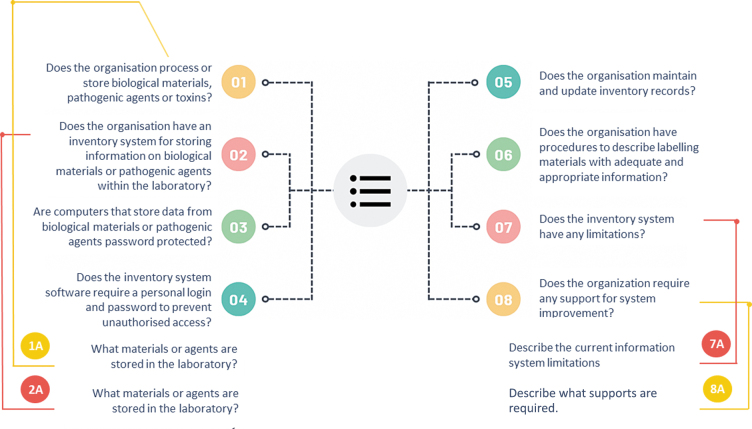
Diagram of the survey questionnaire.

Biological materials and pathogenic agents were stored and handled in all 13 centers. These centers tested various pathogenic agents, ranging from RG1 to RG3. Table 1 summarizes the results of the questionnaire survey, with the left column corresponding to the question number in Figure 1. Table 2 presents confirmed pathogenic materials classified in RG2 and higher. Eight centers processed and stored RG3 agents, ranging between 1 and 1000 aliquots.

**Table 1.** Outcome of data collections

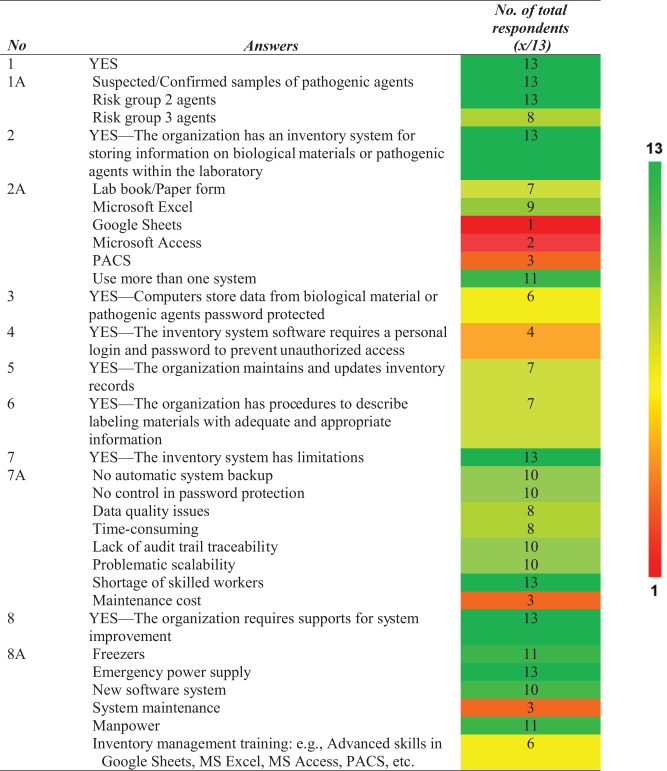

The color range represents the number of respondents: deep green (maximum *n* = 13) to deep red (*n* = 1).

PACS, Pathogen Asset Control System.

**Table 2.** List of risk group 2 and 3 pathogenic materials stored at the veterinary laboratory centers

***Pathogenic species***	***Pathogen type***	***Type of materials stored***	***Pathogen Risk group* ^57,58^**
*Actinomyces* spp.	Bacteria	In vitro isolates	2
*Brucella* spp.	Bacteria	In vitro isolates Specimens from suspected/confirmed cases	3
*Burkholderia pseudomallei*	Bacteria	In vitro isolates	3
*Campylobacter* spp.	Bacteria	In vitro isolates	2
*Enterococcus* spp.	Bacteria	In vitro isolates	2
*Leptospira* spp.	Bacteria	Specimens from suspected/confirmed cases	2
*Salmonella* spp.	Bacteria	In vitro isolates	2
Caprine arthritis encephalitis virus	Virus	Specimens from suspected/confirmed cases	2
Classical swine fever virus	Virus	Specimens from suspected/confirmed cases	2
Equine encephalitis virus	Virus	Specimens from suspected/confirmed cases	3
African swine fever virus	Virus	Specimens from suspected/confirmed cases	3
Foot and mouth disease virus	Virus	In vitro isolates Specimens from suspected/confirmed cases	3
Infectious bronchitis virus	Virus	In vitro isolates	2
Avian influenza virus	Virus	In vitro isolates Specimens from suspected/confirmed cases	3
Newcastle disease virus	Virus	In vitro isolates Specimens from suspected/confirmed cases	2
Porcine reproductive and respiratory syndrome virus	Virus	Specimens from suspected/confirmed cases	2
Rabies virus	Virus	Specimens from suspected/confirmed cases	3

Various inventory systems were used by these laboratories, including paper-based systems (logbooks and paper-based forms), in-house electronic-based systems (Microsoft Excel, Microsoft Access, and Google Sheets), and the Pathogen Asset Control System (PACS). PACS was developed and distributed under the terms of the U.S. Defense Threat Reduction Agency (DTRA)–the U.S. Biological Threat Reduction Program (BTRP) license.

Our data showed that 11 of the 13 laboratory centers used multiple inventory systems, such as logbooks and worksheets in Microsoft Excel. A key outcome from the group discussions was that a few laboratories needed an inventory system for recording material information instead of tracking specimens using handwritten labels on sample tubes.

Reasons for not having a log system included small-scale sample storage and short-term storage due to a laboratory policy for retentions of each type of material. These outcomes reflect a general absence of standard operating procedures (SOP) for biological inventory management. This applies to all entities within the scope of this study, both laboratory centers and government veterinary laboratories.

Only 6 of the 13 centers used an electronic-based system with password access to desktop computers. Computer workstations were used by individual users to access software to perform their work. At the same time, login was required to access biological inventory database software in the laboratory. Four centers had different accounts and password access to the login for computers and inventory systems, whereas the others used the same password for both accesses. Seven centers (*n* = 13) periodically maintained and updated their inventory records and had procedures for labeling stored materials with appropriate and adequate information for intersystem data linking.

**Figure 2.**
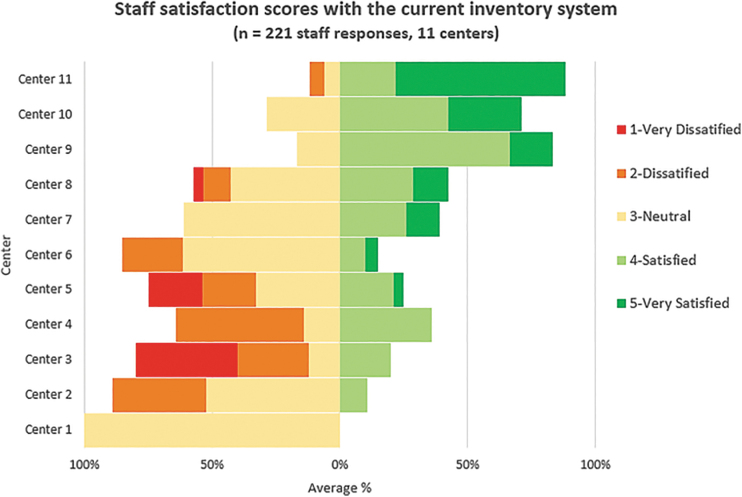
The average percentage of staff satisfaction with their current inventory system from 11 centers in Thailand.

Appropriate and adequate information includes a unique identifier for each item, type of material or pathogen, and date of material collection or production. Common constraints of each inventory system used by these centers are presented in Table 3. A total of 221 staff responses from the 11 centers in Thailand were analyzed to determine average staff satisfaction with the current system (Figure 2).

**Table 3.** Common constraints of each inventory system used by the centers

***Constraints***	***Inventory system of biological materials***				
	***Paper-based***	***Microsoft^®^ Excel***	***Google Sheets***	***Microsoft Access***	***PACS* ^a^**
No automatic system backup	✓	✓	✓	✓	
No user and password verification	✓	✓			
Data quality issues: e.g., mis-spelling, redundancy, inconsistency, different formats, etc.	✓	✓			
Time-consuming	✓	✓			
Lack of auditing trail for traceability of life-cycle details in each record: e.g., identification of user accessioning material into inventory, original quantity accessioned, changes made to quantities in inventory (i.e., removal of material), the reason for the change, individual removing material, etc.	✓	✓	✓	✓	
Problematic scalability	✓	✓	✓	✓	
Skilled-staff shortage	✓	✓	✓	✓	✓
Maintenance costs (i.e., hardware)					✓

^a^ BTRP-DTRA Pathogen Asset Control System.

BTRP-DTRA, U.S. Biological Threat Reduction Program-U.S. Defense Threat Reduction Agency.

Staff scored neutral to low satisfaction in 8 of the 11 centers, whereas 3 centers were mainly satisfied with their existing systems. A total of 46 people from 55 responses who scored higher satisfaction were from laboratories that established electronic-based inventory systems using relational database programs such as PACS and Microsoft Access. In contrast, most low scores were presented from laboratories that used the paper-based system. Gaps identified by our study with recommendations for future improvement of biosecurity are summarized in Table 4.

**Table 4.** Gap identification and recommendations

Gaps	Recommendations
Inventory system: Difficulties of data handling and data tracking when a centralized system was not available, e.g., data redundancy, data inconsistency, limited data sharing, poor data integrity, and difficulty in maintaining information security regarding inventory and data. Chances of data loss and damage where handwriting labels on sample tubes were used for tracking information.	Encourage using an electronic-based system for inventory control in replacement of paper-based systems or handwritten labels.^36^ Develop standard operating procedures that guide and define minimum requirements of an inventory system and types of materials for inventory accounting.^35^ Assign personnel to regularly update inventory records and be responsible for reviewing and approving plans for transferring biological materials to another laboratory and/or disposing of materials from the inventory system.^35,40^ Integrate the use of barcode technology to enhance label integrity, particularly for large-scale studies, which will reduce errors from traditional methods of handwriting labels.^36,37^
Inventory controls and updates: Reporting incidents of inventory discrepancy caused by incomplete inventory updates, e.g., materials were misplaced, not found in storage locations but recorded in the system and vice versa. Cost of staff hours trying to investigate and resolve inventory discrepancies.	Establish procedures for updating inventory records whenever material movements occur.^35,38,40^ Provide regular training for staff on important techniques to maintain inventory continuity and integrity.^35,37^ Define plans for regular inventory audits to review the procedures and identify issues that could cause discrepancies and what improvements are required.^20,22,36,40^ Create a process to respond when inventory discrepancies are identified.^22^ Establish a policy to identify personnel who have authorized access to inventory storage.^22^ Implement a means of securing inventory storage locations.^22^ This could include locks on freezers or the door where freezers are kept, lockboxes inside freezers if the materials are deemed to be highly valuable biological materials (or pose a greater risk for misuse)
Information security: Less than 50% of assessed centers implement data access control through verification of a username and password. Poor security habits pose security risks to electronic information, e.g., using a weak password, password sharing, passwords physically written down near the computer, and leaving the computer without logging off.	Advocate staff awareness of practices that protect sensitive information from unauthorized access.^22^ Support specific training or incorporate programs to respect and value the importance of biological information held in laboratories. This should include information on what data are sensitive, consequences of data release to unauthorized users and concepts of dual-use.^33,35^ Establish a policy to identify personnel who have authorized access to sensitive information held in either computer workstations or backup storage locations.^36^ Implement the use of strong passwords for access verification and complement them with proper security habits training for enhancing awareness of data protection.^39^ Assign staff member(s) the responsibility of continually updating and upgrading antivirus and desktop software to minimize risks from unauthorized access and computer viruses and other malware.^22,39^
Cost management for maintaining inventory: Lack of planning for long-term inventory maintenance and sustainability.	Ensure sufficient funding to sustain long-term storage of specimen inventories, including^36^: Facilities, e.g., adequate storage space for biomaterial retention, a backup power system for a continuous power supply (particularly in areas where power supply is unstable and intermittent), and physical security control to protect the inventory from unauthorized access; Staff training and hiring staff with appropriate expertise, e.g., the cost of recruiting experienced staff, and regular training for maintaining and improving staff skills in inventory management, the cost of time spent on the process of system installation, implementation, maintenance, and sustainability; Software license and maintenance fees if using commercial software.

## Discussion

Establishing a suitable accountability and inventory system is necessary for laboratory biosecurity. These systems control and mitigate risks associated with handling biological agents, particularly from unauthorized access, theft, loss, and misuse.^20,35^ Although a global trend of laboratory biosecurity has been widely considered to be developed and implemented in many laboratories,^41–43^ guidance on inventory controls for pathogenic agents still needs comprehensive detail,^35^ especially for the SEA region.

Access control for high-risk materials is generally not well regulated and identified as a common biosecurity gap in the SEA region,^43^ which leaves the countries vulnerable to possible theft, diversion, or misuse of dangerous pathogens.^44^

Our study collected survey data from government veterinary laboratory centers in Thailand, Lao PDR, and Cambodia. Our survey revealed a diversity of inventory software, a broad range of stored biological materials and pathogens, staff satisfaction scores with the inventory system, typical constraints associated with inventory systems, and support requirements for future improvement in different laboratories. Our study provided tailored recommendations for each gap, taking into account practicality in low and middle-income countries (LMICs).

We used guidance documents,^20,22,35,40^ and best practices from various organizations,^36–38^ and previous publications' recommendations.^33,39^ The guidance documents^20,22,35,40^ provide universal guidelines on biosafety, biosecurity, and biorisk management, including material control and accountability necessary for laboratories housing biological materials.

Recommendations for inventory control include defining what materials exist, where they are stored, which data should be recorded, who has authorized access to the inventory and records, how to manage and update the inventory, who is responsible or the owner, and how to keep records of materials consumed, destroyed, or transferred. Best practices for repositories are shared by various organizations,^36–38^ whereas several instructions are applicable to enhance inventory management, such as considering an inventory system and conducting a periodic audit of the inventory.

These principles are aligned with the WHO guidance, which states, “the biological agent inventory should be up-to-date, complete, accurate and updated regularly to ensure that there is appropriate control and accountability.”^20^ To strengthen the inventory control system, tailor-proposed guidelines to meet laboratory-specific needs are integrated with instructions in Table 4. However, sufficient budget and human resources are required to implement recommendations.

Costs associated with an inventory system include sample handling and processing, storage facilities, security and access control measures, hardware and software purchase and maintenance, staff training, etc.^36,45^ In 2021, the Thai government budgeted to implement a Laboratory Information Management System (LIMS) at all government veterinary laboratory centers.

Like Thailand, Lao PDR and Cambodia's national veterinary laboratories will soon implement an LIMS supported by international organizations.^46,47^ In Lao PDR and Cambodia, external funding supports this implementation and future support for infrastructure upgrades and system maintenance and requires internal funding to achieve long-term sustainability.

Thailand implemented the Pathogen and Animal Toxins Act in 2015 to regulate the use, possession, importation, exportation, and transfer of pathogens and toxins to reduce potential harm to the public.^48,49^ Based on the legislation, laboratories storing dangerous pathogens must annually submit their pathogen inventory to the Department of Medical Science, under the Ministry of Public Health.^48^ They must comply with all requirements to obtain a 1-year national license, granting the laboratory for activities such as the production, possession, transfer, or disposal of pathogens and toxins as specified in the license.^48,50^

Should there be unsafe incidences occurring in a laboratory that endanger staff, such as a laboratory-acquired infection, or the public, animals, or the environment through an accidental pathogen escape from a laboratory setting (APELS), the entity must immediately notify the responsible government agency and report the nature of the incident, level of severity, and the quantities of the pathogen.

The responsible agency or designation of other agencies has a right to cease the production and operation processes or conduct inspection or evaluation in case of emergency events.^48^ The findings of the review may result in the notification of restriction or prohibition of the possession, production, or transfer of pathogens by the entity.

In contrast, Lao PDR and Cambodia do not have legislation to regulate biological materials and pathogen inventories. The growing threats of emerging and re-emerging diseases highlight the need for national and regional control mechanisms to enhance biosecurity in SEA. Without a country-specific list of controlled agents, the region lacks a sufficient foundation for oversight, making it vulnerable and unprepared to respond effectively to public health emergencies in a coordinated manner.^32^

Thus, an up-to-date national and regional pathogen inventory means to secure the inventories, and the development of emergency operations for rapid response to disease outbreaks or APELS is crucial for countries to control dangerous pathogens, as described in a recent report from Thailand.^50^ The report compared the development of the Pathogen and Animal Toxin Act 2015 to laws in Canada, the United States, and Singapore.

Multiple inventory systems in laboratory centers make creating an up-to-date institutional database difficult and increases the likelihood of data discrepancies. The lack of a centralized inventory system poses a challenge for laboratories to maintain and synchronize consistent information across multiple systems. We recommend that laboratories establish a centralized inventory system to improve data quality and increase the access control's effectiveness for data security.

Centralized management enables laboratories to securely store and manage data with established protocols, reducing unauthorized access and discrepancies.^37,38^ Successful implementation of a centralized system will require selecting an appropriate inventory system software, a running budget, adequate facilities, equipment, and human resources.^37^

Thailand and other countries can establish a national or regional inventory database by developing accurate institutional inventories. Most regional veterinary laboratories in Thailand only process serum for low-risk diagnostic testing, such as ELISA, and do not work with or store RG3 pathogens. These laboratories often create their biological inventory databases using spreadsheet programs and establish appropriate practical procedures for system management and control.

However, if laboratories later work with biological samples containing RG3 pathogens with the potential to cause severe disease and death, increased safety and control measures will be necessary to prevent harm to people and the environment.

The detailed inventory information should be maintained in the access control system.^51,52^ The COVID-19 pandemic represents an opportunity to centralize inventory management at the national and regional levels. Many laboratory inventory management software and LIMS have been developed for biorepositories and biobanks in laboratory areas.^53,54^

To ensure widespread acceptance of such infrastructure, it would be ideal to lower the initial software license and maintenance fees in LMICs. A no-license fee software, such as DTRA-BTRP supported PACS, is provided to key laboratories supporting human and animal health in SEA due to limited financial resources in LMICs.^55^

An affordable inventory system such as Microsoft Excel could be implemented, but specific SOPs must be established. This includes identifying personnel with authorized access and defining procedures for regular inventory updates.^22,35^

There are limitations to this study. The study only collected data from government veterinary centers in Cambodia and Lao PDR at the national level and both the national and regional levels in Thailand. Therefore, it reflects a limited number of pathogen inventories in the region. Second, staff satisfaction scores were not evaluated at the Lao PDR and Cambodia centers because of low participant numbers during the follow-up group discussions.

Third, as part of the institutional security plan, some data were sensitive and confidential and could not be shared (i.e., lists of pathogen stocks). This issue was a barrier to data collection, as reported in other studies.^56^ Information on handling animal specimens with suspected or confirmed cases of SAR-CoV-2 was not available, and private veterinary hospitals in Thailand, rather than government laboratories, investigated and reported cases of natural infection in animals.^6,7^

Regarding limitations, future studies should include selected research laboratories, those associated with medical and veterinary schools, and university laboratories to fully understand the biosecurity gaps in SEA laboratories.

There is no standard for pathogen inventory and control systems in the region, and it is suggested that laboratories could use recommendations from this report to develop or improve their systems. The guidance offers flexible advice that can be adapted to meet institution-specific requirements for biosafety and biosecurity. It is crucial to establish procedures for regularly updating inventory, conducting periodic inventory audits, and responding to any discrepancies.^20,22,40^

The proposed guidelines aim at addressing significant inventory discrepancies, often recognized in laboratories that lack proper control and updates, intending to strengthen biosecurity and biorepository systems. National and regional efforts to control biorisk and perform scientific studies require coordination and collaboration between human and animal public health laboratories.

Governments play a significant role in setting standards and regulations to enhance the country's biosecurity. They are responsible for providing necessary laboratory support to improve and maintain inventory management systems and controls.

## Authors' Disclosure Statement

No competing financial interests exist.

## Funding Information

The Veterinary Laboratory Capacity Building in Thailand and The Enhancement of Zoonotic Disease Outbreak Detection in Lao PDR and Cambodia projects are a collaboration between Thailand, Lao PDR, and Cambodia and the Mahidol Oxford Tropical Medicine Research Unit (MORU) funded by the Biological Threat Reduction Program of the Defense Threat Reduction Agency (BTRP-DTRA) of the U.S. Government [contract numbers HDTRA1-17-C-0007, and HDTRA1-21-C0047]. This research was funded in whole, or in part, by the Wellcome Trust [220211]. For the purpose of Open Access, the author has applied a CC BY public copyright licence to any Author Accepted Manuscript version arising from this submission.
